# Comprehensive characterization of ERV-K (HML-8) in the chimpanzee genome revealed less genomic activity than humans

**DOI:** 10.3389/fcimb.2024.1349046

**Published:** 2024-02-22

**Authors:** Chunlei Wang, Xiuli Zhai, Shibo Wang, Bohan Zhang, Caiqin Yang, Yanmei Song, Hanping Li, Yongjian Liu, Jingwan Han, Xiaolin Wang, Jingyun Li, Mingyue Chen, Lei Jia, Lin Li

**Affiliations:** ^1^ Department of Microbiology, School of Basic Medicine, Anhui Medical University, Hefei, Anhui, China; ^2^ Department of Virology, Beijing Institute of Microbiology and Epidemiology, Beijing, China; ^3^ State Key Laboratory of Pathogen and Biosecurity, Beijing, China; ^4^ National 111 Center for Cellular Regulation and Molecular Pharmaceutics, Key Laboratory of Fermentation Engineering, Hubei University of Technology, Wuhan, Hubei, China

**Keywords:** endogenous retroviruses, chimpanzee, human, characterization, evolution

## Abstract

Endogenous retroviruses (ERVs) originate from ancestral germline infections caused by exogenous retroviruses. Throughout evolution, they have become fixed within the genome of the animals into which they were integrated. As ERV elements coevolve with the host, they are normally epigenetically silenced and can become upregulated in a series of physiological and pathological processes. Generally, a detailed ERV profile in the host genome is critical for understanding the evolutionary history and functional performance of the host genome. We previously characterized and cataloged all the ERV-K subtype HML-8 loci in the human genome; however, this has not been done for the chimpanzee, the nearest living relative of humans. In this study, we aimed to catalog and characterize the integration of HML-8 in the chimpanzee genome and compare it with the integration of HML-8 in the human genome. We analyzed the integration of HML-8 and found that HML-8 pervasively invaded the chimpanzee genome. A total of 76 proviral elements were characterized on 23/24 chromosomes, including detailed elements distribution, structure, phylogeny, integration time, and their potential to regulate adjacent genes. The incomplete structure of HML-8 proviral LTRs will undoubtedly affect their activity. Moreover, the results indicated that HML-8 integration occurred before the divergence between humans and chimpanzees. Furthermore, chimpanzees include more HML-8 proviral elements (76 vs. 40) and fewer solo long terminal repeats (LTR) (0 vs. 5) than humans. These results suggested that chimpanzee genome activity is less than the human genome and that humans may have a better ability to shape and screen integrated proviral elements. Our work is informative in both an evolutionary and a functional context for ERVs.

## 1 Introduction

Endogenous retroviruses have played a role in primate evolution and result from exogenous retroviral infections, which integrate into the genome of the host germline and are subsequently inherited by the next generation (Stoye, 2012; Mager and Stoye, 2015; Jansz and Faulkner, 2021). ERVs can be found in all vertebrate genomes (Stoye, 2012; Jansz and Faulkner, 2021). For human endogenous retroviruses (HERVs), all residual components of HERVs have accounted for approximately 8% of the whole human genome (Venter et al., 2001; Jia et al., 2022; Liu et al., 2023). The proviral genome consists of a long terminal repeat (LTR) at both ends and four internal open reading frames. The LTRs at both ends contain functional regulatory elements, such as promoters, enhancers, and transcription factor-binding sites (Bannert and Kurth, 2004). The *gag* gene encodes structural proteins of the virus, including matrix (MA), capsid (CA), and nucleocapsid protein (NC). MA forms layer on the inside of the viral envelope and play important roles in virus assembly, as they form links or bridge between nucleocapsids/cores and the envelope. CA is the major structural component and plays a key role in the viral assembly and budding processes. NC is a small zinc finger protein that possesses nucleic acid chaperone activity that enables NC to rearrange DNA and RNA molecules into the most thermodynamically stable structures. The *pro* gene encodes a protease playing a central role in proteolytic processing. The *pol* gene encodes open reading frames for the proteins reverse transcriptase (RT) and integrase (IN). RT is responsible for converting RNA into complementary DNA, a key step in retrovirus replication. IN mediates the insertion of ERVs into the genome of the host cell. The *env* gene encodes surface and transmembrane proteins that participate in the assembly of retrovirus-like particles (Ono, 1986). Many of the coding regions of proviruses have lost the ability to encode functional proteins due to mutations, insertions, deletions, and rearrangements. In addition, the proviruses occasionally undergo homologous recombination between ancestral 5’ and 3’ proviral LTRs, where the intervening protein-coding sequence is deleted to form a separate solitary (or “solo”) LTR. It was reported that at least 85% of ERV cases are solitary (or “solo”) LTRs (Lander et al., 2001; Mager and Stoye, 2015). Surprisingly, there are few similarities between the LTRs of retroviruses from different genera (Benachenhou et al., 2013; Johnson, 2019).

There are many types of ERVs which can be classified according to their phylogenetic relationships. The three main categories are: Class I represents γ retrovirus-like elements, Class II represents β retrovirus-like elements, and Class III represents spuma virus-like elements (Vargiu et al., 2016). The ERV-K group, which belongs to Class II, contains 11 subtypes, which are called Human MMTV Like, so they are named HML with a number (HML1-11). The ERV-K is the most studied group (Barbulescu et al., 1999). In addition to HML-2, HML-6, HML-7, HML-8, and HML-9 have also attracted the attention of many researchers (Lavie et al., 2004; Flockerzi et al., 2005; Broecker et al., 2016; Scognamiglio et al., 2022).

Most sequences of ERVs have been mutated and inactivated, but some ERVs can still be expressed and play important roles in some physiological processes. Studies have shown that ERV transcription occurs in healthy cells and tissues, including embryos and placentas (Stoye, 2012). In addition, aberrant expression of ERVs occurs in several diseases, such as multiple sclerosis and breast cancer, and their proteins may contribute to disease etiology (Jansz and Faulkner, 2021). It has been reported that HERV-K (HML-2) is a risk factor for multiple sclerosis (Garcia-Montojo et al., 2018; Holloway et al., 2019). In addition, the transcription level of ERV is increased in breast cancer, teratoma, ovarian tumor, and melanoma (Garcia-Montojo et al., 2018; Johnson, 2019; Jansz and Faulkner, 2021; Chen et al., 2022; Jia et al., 2022; Liu et al., 2023). In summary, although many ERVs have acquired mutations and are not actively expressed, there are ERV loci that continue to have important biological functions.

Therefore, considering the substantial contribution of ERVs to the host genome and their emerging roles in shaping the host’s regulatory networks, exploring the dynamic expression and function of ERVs is important for understanding both human- and primate-specific aspects of gene regulation and development, including physiological and pathological processes (Kunarso et al., 2010; Grow et al., 2015). Before the dynamics of ERVs can be examined, it is essential to first determine the distribution and position of ERVs in the host genome. Many studies have focused on ERV elements in the human genome, but only a few have concentrated on these elements within the nonhuman primate genome. For chimpanzees, which are the closest living relative of humans, previous work revealed 45 HML-2 elements inserted specifically into the chimpanzee genome (Macfarlane and Badge, 2015). The results indicated that, compared with humans, the chimpanzee genome contains less chimpanzee-specific HML-2 integration. In addition, little work has been done to characterize ERVs in chimpanzees and compare these with those of other primates, such as gorillas and humans (Holloway et al., 2019). Previously, we performed comprehensive identification and characterization of the ERV-K (HML-8) group in the human genome (Liu et al., 2023). However, the distribution and function of HML-8 elements in other primates, such as chimpanzees remain unclear, and comparisons of the genomic distribution, integration time, and potential regulatory roles between the two hosts have not been performed. Chimpanzees are the closest living relative of human beings (Bannert and Kurth, 2006). Therefore, accurate and complete characterization of HML-8 elements in the chimpanzee genome is needed to compare the evolutionary forces underlying the 2 recent speciation patterns of mammalian groups. This work will facilitate the study of the existence, evolutionary relationship, and function of ERVs in primates, potentially helping to elucidate the pathogenesis of serious human diseases.

## 2 Materials and methods

### 21 HML-8 identification, localization, and genomic distribution

We used Jan. 2018 (Clint_PTRv2/panTro6) as the chimpanzee reference genome to determine the distribution of HML-8 remnants in the chimpanzee genome. The assembled MER11A-HERVK11-MER11A sequence from the Dfam database was used as a query for the HML-8 reference (Hubley et al., 2016) (https://dfam.org/home). There are typically two resources for reference: consensus representatives and single best representatives. Compared to the single best representative, which is a specific and high-quality ERV sequence for HML-8, the consensus sequence for HML-8 has a much broader representation. Therefore, consensus representatives are used as references or queries in most studies (Grandi et al., 2017; Pisano et al., 2019). The BLAT search tool in the UCSC genome browser database was used to detect the integrated HML-8 elements (Kent, 2002; Kent et al., 2002). BLAT on DNA is designed to quickly find sequences of 95% and greater similarity of length 25 bases or more. BLAT functions in DNA alignment by keeping an index of the entire genome in its memory. The index consists of all overlapping 11-mers stepping by 5 except for those heavily involved in repeats (http://genome.ucsc.edu/cgi-bin/hgBlat). Additionally, the expected distribution of HML-8 loci on each chromosome was calculated according to the Formula e=Cl × N/Tl (e is the expected integration number in the chromosome, Cl represents the nongap length of the chromosome, N represents the total number of actual HML-8 loci identified in the human genome, and Tl represents the total nongap length of all chromosomes) (Grandi et al., 2021; Jia et al., 2022; Liu et al., 2023). Chi-square (χ2) tests were performed to analyze the difference between the expected integration number and the actual number of HML-8 loci and to estimate the statistical significance based on the *p* value.

### 2.2 Structural characterization

The length and structure of all the HML-8 provirus remnants were characterized via multiple alignments with the Dfam reference MER11A-HERVK11-MER11A performed with MEGA 7 and the BioEdit software platform (Kumar et al., 2016; Tamura et al., 2021). All the structural details, including insertions and deletions, were annotated.

### 2.3 Phylogenetic analyses

To confirm the assignment of the identified HML-8 elements in the chimpanzee genome, maximum likelihood (ML) phylogenetic trees were constructed using MEGA 7 (Kumar et al., 2016). Elements containing many gaps were eliminated manually. Three proviral sequences (longer than 80% of the HML-8 reference length) were screened to determine their phylogenetic relationships. Using the model selection function in MEGA7, the best-fit model of nucleotide substitution for these near full-length proviruses was the general time reversible model with a gamma distribution and invariant sites (GTR+G+I). Additionally, elements longer than 90% of the corresponding 4 coding regions of HML-8 were screened to construct subregion phylogenetic trees, respectively. Based on the model selection model in MEGA7, the most suitable nucleotide substitution models for *gag*, *pro*, *pol* and *env* analysis are as follows: the Hasegawa-Kishino-Yano model with a gamma distribution and invariant sites (HKY+G+I); the general time reversible model with a gamma distribution and invariant sites (GTR+G+I); the general time reversible model with a gamma distribution (GTR+G); and the Hasegawa-Kishino-Yano model with a gamma distribution (HKY+G). The nearest neighbor interchange (NNI) procedure was used to search for the tree topology. The nearest neighbor interchange is a heuristic search to improve the likelihood of a tree by performing the following operation on it. If we have two unrooted trees, then we can specify a neighbor relation between the two of them and then swap their subtrees in an attempt to obtain a tree that has a higher likelihood (https://www.megasoftware.net/webhelp/centraldialogbox_hc/nearest_neighbor_interchange_nni_.htm). The confidence of each node in the phylogenetic trees was determined using the bootstrap test with 500 bootstrap replicates. The final trees were visualized by iTOL (Tamura et al., 2021).

### 2.4 Estimation of the integration time of HML-8 members in the chimpanzee genome

To estimate the integration time of each HML-8 element in the chimpanzee genome, we used a substitution rate of 0.2%/nucleotide/million years to evaluate the divergence effect on every HML-8 (Lebedev et al., 2000). For the 4 internal regions (*gag*, *pro*, *pol*, and *env*), the integration time was calculated based on the Formula T = D/0.2. For the flanking LTR regions, the integration time was calculated based on the Formula T = D/0.2/2. T represents the estimated time of integration (in million years). D represents the percentage of divergent nucleotides, and the D of each HML-8 element was estimated in two ways: (1) between the 5’ and 3’ LTRs of each provirus and (2) between each HML-8 internal element and its consensus generated. The divergence values were calculated with MEGA7.

### 2.5 Functional prediction of cis-regulatory regions and enrichment analysis

The noncoding LTR regions of HML-8 lack biological function annotations in the chimpanzee genome. To understand the biological significance of the HML-8 proviral LTRs, we performed functional prediction and enrichment analysis of the cis-regulatory regions of these HML-8 chimpanzees. Based on the Genomic Regions Enrichment of Annotations Tool (GREAT), gene annotations near HML-8 proviral LTRs were analyzed. The association rules were as follows: basal + extension, 5000 bp upstream, 1000 bp downstream, and 1000000 bp maximum extension; curated regulatory domains were included. When the potential regulatory genes were identified, the WEB-based Gene SeT Analysis Toolkit (WebGestalt) was subsequently used to analyze their functional enrichment (http://www.webge stage). org). This approach is crucial for interpreting the list of genes of interest. The enrichment method used here was overrepresentation analysis (ORA). The parameters for the enrichment analysis included the following: minimum number of IDs in the category: 5; maximum number of IDs in the category: 2000; FDR Method: Benjamini–Hochberg (BH); and significance level: top 10.

## 3 Results

### 3.1 Identification, localization, and distribution of HML-8 remnants in the chimpanzee genome [Jan.2018 (Clint_PTRv2/panTro6)]

The results showed that HML-8 elements pervasively invaded the chimpanzee genome. According to the BLAT results obtained for MER11A-HERVK11-MER11A in Jan. 2018 (Clint_PTRv2/panTro6), we identified a total of 76 HML-8 proviral elements (**Table 1**), as compared to the 40 proviral elements we identified in the human genome (Liu et al., 2023). Based on the integrated genomic loci, each HML-8 element was named according to the nomenclature previously proposed for HERV-K elements (**Table 1**) (Subramanian et al., 2011). First, we observed a notable feature: there was no complete full-length element of HML-8 in the chimpanzee genome. The longest proviral element was 9158 bp long, which accounted for 84.7% of the reference sequence. The length analysis revealed that the average length of these proviral elements was 4378 bp, with 9 elements being greater than 70% of the reference length, 21 elements being between 40 and 70% of the reference length, and the remaining 46 elements being between 8.14 and 37.49% (**Table 1**). Among them, the shortest proviral element was 875 bp long, which accounted for only 8.14% of the reference sequence. The longest and shortest HML-8 proviral elements in the human genome are 9162 and 874, respectively. This similarity suggested that the integration events of HML-8 simultaneously occurred before the divergence between humans and chimpanzees.

**Table 1:** HML-8 provirus distribution.

Number	Chromosome	Strand	Position start	Position end	Length (bp)	Match+mismatch(bp)/full length(bp)	Range	Qgap(bp)/match+mismatch+Qgap(bp)	Insertion or deletion	Intergenic/intron/exon
1	chr11	–	97063674	97072831	9158	84.70%	【80%-90%)	3.28%	NA	intergenic
2	chr19	–	23582963	23597406	14444	81.45%	【80%-90%)	5.78%	Insertion	intergenic
3	chr17	+	28556159	28565079	8921	81.22%	【80%-90%)	9.29%	NA	intergenic
4	chr1	+	156345936	156354251	8316	76.67%	【70%-80%)	12.10%	Deletion	intergenic
5	chr9	+	31695596	31703805	8210	76.57%	【70%-80%)	2.80%	NA	intergenic
6	chr5	–	52655093	52662923	7831	73.29%	【70%-80%)	4.76%	NA	intergenic
7	chr19	–	25615095	25622844	7750	71.85%	【70%-80%)	6.43%	NA	intergenic
8	chr12	–	51714625	51722440	7816	70.94%	【70%-80%)	7.63%	NA	intergenic
9	chr6	–	73941843	73949302	7460	70.49%	【70%-80%)	4.11%	NA	intergenic
10	chr9	–	84591713	84599232	7520	69.51%	【60%-70%)	24.21%	Deletion	intron
11	chr3	+	79615035	79622061	7027	64.57%	【60%-70%)	17.82%	Deletion	intron
12	chrX	+	56602551	56609242	6692	63.26%	【60%-70%)	4.37%	NA	intergenic
13	chr1	–	135696367	135712562	16196	61.48%	【60%-70%)	6.97%	Insertion	intron
14	chr11	+	63656712	63663509	6798	59.90%	【50%-60%)	31.36%	Deletion	intergenic
15	chr2A	+	64123614	64129736	6123	57.81%	【50%-60%)	20.10%	Deletion	intron
16	chr3	–	128565266	128571536	6271	57.64%	【50%-60%)	25.16%	Deletion	intron
17	chr12	–	81696811	81702819	6009	56.24%	【50%-60%)	13.24%	Deletion	intergenic
18	chr11	+	49590235	49596119	5885	54.38%	【50%-60%)	5.42%	NA	intergenic
19	chr10	–	98677603	98683109	5507	50.71%	【50%-60%)	37.81%	Deletion	genic &intergenic
20	chr1	+	108430301	108435591	5291	49.26%	【40%-50%)	32.31%	Deletion	intergenic
21	chrY	+	23381750	23386956	5207	48.45%	【40%-50%)	31.40%	Deletion	intergenic
22	chr11	+	50352478	50357771	5294	48.21%	【40%-50%)	9.03%	NA	intergenic
23	chr11	–	49637737	49642530	4794	43.57%	【40%-50%)	35.29%	Deletion	intergenic
24	chr4	+	137449286	137454131	4846	42.39%	【40%-50%)	45.12%	Deletion	intergenic
25	chr12	+	102968149	102972730	4582	42.14%	【40%-50%)	8.05%	NA	intergenic
26	chr3	+	109740319	109744759	4441	41.74%	【40%-50%)	30.22%	Deletion	intergenic
27	chr11	–	14869711	14874159	4449	41.19%	【40%-50%)	26.33%	Deletion	intergenic
28	chr4	+	64843173	64847502	4330	40.68%	【40%-50%)	3.57%	NA	intergenic
29	chrX	+	34789238	34793597	4360	40.26%	【40%-50%)	24.69%	Deletion	intergenic
30	chr8	+	44511870	44516437	4568	40.01%	【40%-50%)	7.33%	NA	intergenic
31	chr6	–	155777868	155781847	3980	37.49%	【30%-40%)	1.50%	NA	exonic&intronic
32	chr19	+	24073629	24078094	4466	36.96%	【30%-40%)	4.79%	NA	intergenic
33	chr4	–	77803811	77807756	3946	36.44%	【30%-40%)	34.61%	Deletion	intergenic
34	chr8	–	12333790	12337817	4028	36.18%	【30%-40%)	27.03%	Deletion	intron
35	chr7	+	6075204	6079128	3925	35.29%	【30%-40%)	28.35%	Deletion	intron
36	chr8	+	43749874	43753733	3860	34.71%	【30%-40%)	38.35%	Deletion	intergenic
37	chrY	+	6360429	6370254	9826	34.32%	【30%-40%)	38.19%	Insertion,Deletion	intergenic
38	chr8	–	86619640	86623356	3717	34.09%	【30%-40%)	34.77%	Deletion	intergenic
39	chr20	+	29240803	29250628	9826	34.03%	【30%-40%)	38.72%	Insertion,Deletion	intergenic
40	chr4	+	77195305	77198763	3459	32.47%	【30%-40%)	4.25%	NA	intergenic
41	chr2A	+	102337670	102341003	3334	31.08%	【30%-40%)	11.22%	Deletion	intergenic
42	chr5	+	150548523	150551566	3044	28.84%	【20%-30%)	28.68%	Deletion	intron
43	chr7	+	50863865	50867270	3406	27.87%	【20%-30%)	28.99%	Deletion	intergenic
44	chr1	+	45743923	45746943	3021	27.13%	【20%-30%)	10.68%	Deletion	intergenic
45	chrX	+	41627863	41630750	2888	26.01%	【20%-30%)	13.10%	Deletion	intron
46	chrX	–	42154219	42156882	2664	24.40%	【20%-30%)	13.70%	Deletion	intergenic
47	chr22	–	1446161	1448721	2561	23.70%	【20%-30%)	1.58%	NA	intron
48	chr16	–	44723847	44726410	2564	23.53%	【20%-30%)	11.83%	Deletion	intergenic
49	chrY	–	3095532	3098114	2583	23.20%	【20%-30%)	14.79%	Deletion	intergenic
50	chrY	+	13438427	13440964	2538	23.15%	【20%-30%)	13.88%	Deletion	intergenic
51	chr19	–	20847619	20850034	2416	22.88%	【20%-30%)	2.80%	NA	intergenic
52	chr8	–	11324731	11327317	2587	22.77%	【20%-30%)	15.32%	Deletion	intergenic
53	chr1	+	31825639	31827932	2294	21.66%	【20%-30%)	44.57%	Deletion	intergenic
54	chr2B	–	5807936	5810201	2266	21.54%	【20%-30%)	5.44%	NA	intergenic
55	chr4	+	49398166	49400489	2324	21.00%	【20%-30%)	14.29%	Deletion	intergenic
56	chr15	–	19096168	19099630	3463	20.90%	【20%-30%)	21.64%	Insertion,Deletion	genic&intergenic
57	chr4	+	162706670	162708848	2179	20.70%	【20%-30%)	5.49%	NA	intergenic
58	chr2B	+	100074966	100077139	2174	20.56%	【20%-30%)	5.31%	NA	intron
59	chr18	–	44459897	44461968	2072	19.48%	【10%-20%)	0.54%	NA	intergenic
60	chr19	+	25500990	25503068	2079	19.47%	【10%-20%)	2.58%	NA	intergenic
61	chr14	+	71784651	71786596	1946	18.45%	【10%-20%)	8.25%	NA	intron
62	chr4	+	55246241	55248103	1863	17.68%	【10%-20%)	10.87%	Deletion	intergenic
63	chr6	+	58293089	58294835	1747	16.57%	【10%-20%)	0.63%	NA	intergenic
64	chr13	–	59301174	59302916	1743	16.29%	【10%-20%)	6.77%	NA	intergenic
65	chr11	–	49783110	49784802	1693	15.89%	【10%-20%)	2.00%	NA	intergenic
66	chr2A	–	77283713	77285331	1619	15.17%	【10%-20%)	1.55%	NA	intron
67	chr2B	–	97034956	97036587	1632	15.12%	【10%-20%)	14.74%	Deletion	intergenic
68	chr5_NW_019932883v1_random	–	1485086	1486708	1623	14.83%	【10%-20%)	17.16%	Deletion	intergenic
69	chr4	–	50892584	50893971	1388	12.91%	【10%-20%)	15.64%	Deletion	intron
70	chrY	–	14091617	14092838	1222	11.28%	【10%-20%)	4.13%	NA	intergenic
71	chrY	–	5451943	5453164	1222	11.28%	【10%-20%)	4.13%	NA	intergenic
72	chrX	–	46463406	46464551	1146	10.90%	【10%-20%)	16.33%	Deletion	intergenic
73	chrY	–	1087726	1088806	1081	10.28%	【10%-20%)	0.83%	NA	intergenic
74	chr5	–	32949251	32950412	1162	9.90%	【0%-10%)	11.51%	Deletion	intergenic
75	chr10	–	77164804	77165954	1151	9.65%	【0%-10%)	13.06%	Deletion	intergenic
76	chr1	+	104828192	104829066	875	8.14%	【0%-10%)	2.18%	NA	intergenic

“-” indicates antisense strand, “+” represents sense strand, and “NA” stands for Not Applicable.

**Figure 1:**
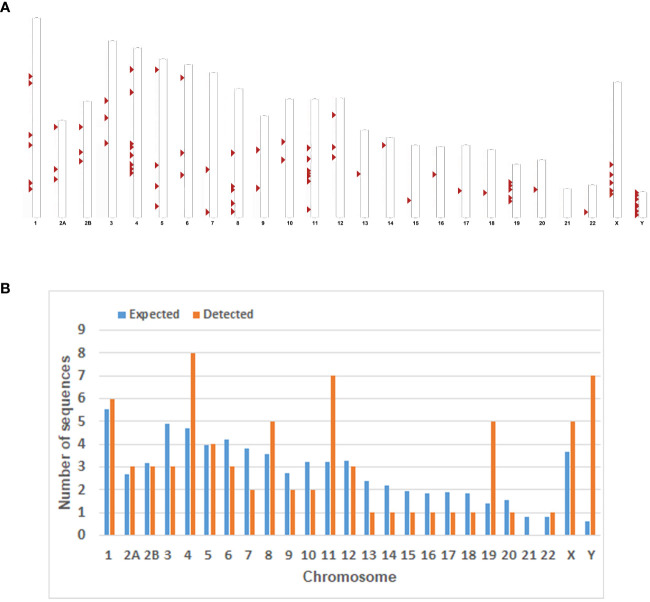
Chromosomal distribution of the HML-8 loci in the chimpanzee genome. **(A)** All the HML-8 elements (red arrows) are displayed on the chimpanzee karyotype (http://www.ensembl.org). **(B)** The number of HML-8 proviral elements integrated into each Chimpanzee chromosome was determined and compared to the expected number of insertion events. The expected number of sequences in each chromosome is marked in blue, and the actual number of detected sequences is marked in orange.

We did not find any solo HML-8 LTRs in the chimpanzee genome which is distinct from our findings in the human genome where there were 5 solo HML-8 LTRs. Although being short (approximately 75% of the representative reference MER11A), 5 solo LTRs exist in human genome. The nucleotide sequence of each proviral element is shown in **Supplementary Dataset 1**. The underlying distribution of HML-8 elements in the chimpanzee genome is shown in **Figure 1A**.

Furthermore, the expected number of HML-8 proviral elements in each chimpanzee chromosome was predicted. The expected number of HML-8 loci was subsequently compared with the actual number of HML-8 loci detected on each chimpanzee chromosome to evaluate whether HML-8 was randomly distributed in the chimpanzee genome. The results indicated that the number of observed HML-8 distribution events was significantly inconsistent with what was expected, thus supporting the nonrandom integration of HML-8 in the genome (**Figure 1B**). For proviral elements, the number of HML-8 insertions on chromosomes 4, 11, 19, and Y was greater than expected. In particular, the number of HML-8 proviral elements on the Y chromosome was 12 times greater than expected. In contrast, on chromosomes 3, 6, 7, 9, 10, 13, 14, 15, 16, 17, 18 and 20, the number of HML-8 locus integrations was lower than expected. Notably, we did not detect any HML-8 proviral integrations on chromosome 21 (**Figure 1B**). The analysis clearly showed that the integration of HML-8 into the chimpanzee genome was nonrandom. Furthermore, all 76 identified proviral elements were analyzed to determine their locations in intergenic regions, introns, or exons. The results showed that 59 proviral elements were located in intergenic regions, accounting for 77.63%; 14 proviral elements were located in introns, accounting for 18.42%; 2 proviral elements were located in both genic and intergenic regions, accounting for 2.63% (**Table 1**). Brady et al. previously validated that the accumulation of HML-2 proviruses in introns and intergenic regions is a selection against proviruses that integrate into exons and genic regions rather than a result of integration preference (Brady et al., 2009). Our study similarly revealed a nonrandom distribution and apparent bias for insertions into intergenic regions and introns.

### 3.2 Structural characterization

The analysis of the structural features of all 76 HML-8 proviruses, such as deletion and insertion events, can characterize the uniqueness of each proviral element and assess the potential for active expression. Thus, to define the structural characteristics of HML-8, the 76 proviral elements were first compared to the complete HML-8 reference (MER11A-HERVK11-MER11A). According to the annotation information in the Dfam database (https://www.dfam.org/family/DF0000193/features), the complete HML-8 reference exhibited a typical proviral structure containing 4 open reading frames (ORFs) and 2 flanking LTRs. Specifically, the 5’ LTR is located between nucleotides 1-1266, the coding sequence (CDS) range of the HERVK11 *gag* protein is from nucleotides 1422-3530, the CDS range of the HERVK11 *pro* protein is from nucleotides 3341-4345, the CDS range of the HERVK11 *pol* protein is from nucleotides 4303-7032, the CDS range of the HERVK11 *env* protein is from nucleotides 6890-9217, and the 3’ LTR is located between nucleotides 9220-10485.

**Figure 2:**
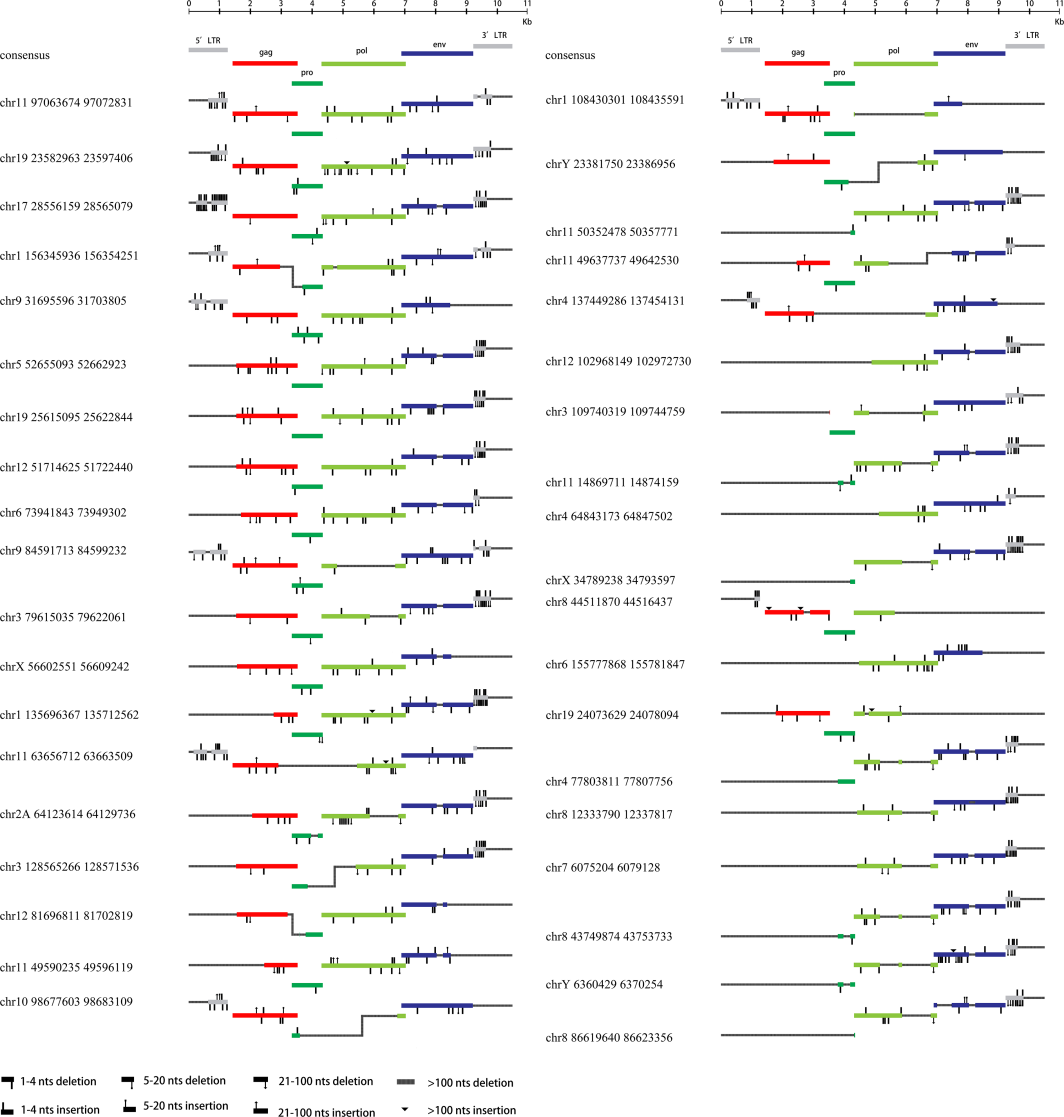
HML-8 proviruses structural characterization of elements 1-38. The front (1-38) HML-8 provirus elements were analyzed and compared with the Dfam reference sequence. All insertions and deletions have been annotated, as reported in the figure legend. The way the loci were grouped depended on the range of their sequence match to the consensus sequence.

**Figure 3:**
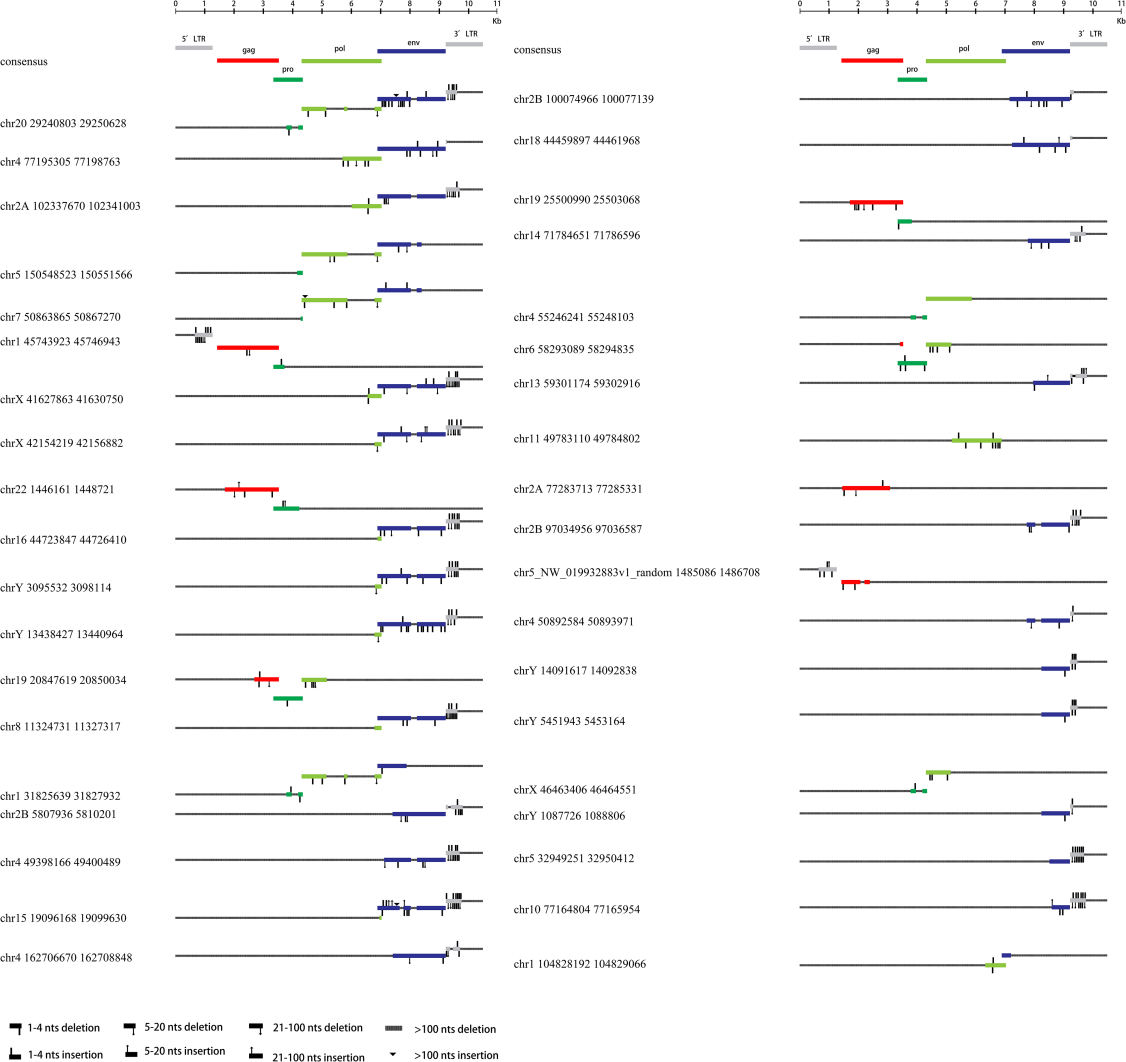
HML-8 proviruses structural characterization of elements 39-76. The following (39-76) HML-8 provirus elements were analyzed and compared with the Dfam reference sequence. All insertions and deletions have been annotated, as reported in the figure legend. The way the loci were grouped depended on the range of their sequence match to the consensus sequence.

All 76 HML-8 proviral sequences were aligned, and the positions of the insertions and deletions were annotated to describe the structure of each HML-8 provirus element (**Figures 2**, **3**). We grouped HML-8 proviral loci based on their alignment to the consensus sequence. We found that all HML-8 loci in the chimpanzee genome were incomplete and lacked either some part of an LTR, internal coding sequences, or both. Among them, only 9 elements, including HML-8 chr11:97063674-97072831, chr19: 23582963-23597406, chr17:28556159-28565079, chr1:156345936-156354251, chr9:31695596-31703805, chr5:52655093-52662923, chr19:25615095-25622844, chr12:51714625-51722440, and chr6:73941843-73949302, were longer than 70% of the complete reference sequence in length and showed the typical proviral structure (**Figure 2**).

Additionally, **Table 2** summarizes the integrity of the 6 separate regions relative to the corresponding sections of the HML-8 reference sequence (5’ LTR, *gag*, *pro*, *pol*, *env*, and 3’ LTR), respectively. The results showed that among all 76 proviral elements, the 5’ LTR regions of 63 were missing. The longest 5’ LTR included 1023 base pairs out of 1266 base pairs (80.81%) relative to the corresponding reference region. The shortest 5’ LTR included 179 base pairs out of 1266 base pairs (14.14%). The remaining 11 5’ LTRs ranged from 33.49%-76.54% (**Table 2**). The 5’ LTR plays a crucial role in virus transcription and replication. Due to the truncation of the HML-8 proviral LTR sequences, it is unlikely these proviruses are actively expressed or able to retrotranspose into new locations in the chimpanzee genome. Among all 76 proviral elements, 43 *gag* regions have been deleted. The shortest *gag* gene accounts for 0.52%. The 15 *gag* loci range from 90.04%-99.72%. The remaining 17 *gag* loci ranged from 5.22%-86.58% (**Table 2**). Among all 76 proviral elements, 34 *pro* regions have been deleted. The shortest *pro* gene accounts for 3.28%. The 17 *pro* loci ranged from 91.84%-99.90%. The remaining 22 *pro* loci ranged from 7.06%-88.06% (**Table 2**). Among all 76 proviral elements, 19 *pol* regions have been deleted. The shortest *pol* gene accounted for 2.67%. The 15 *pol* loci range from 92.89%-99.82%. The remaining 41 *pol* loci ranged from 5.13%-78.46%. Among all 76 proviral elements, 11 *env* regions have been deleted. The shortest *env* gene accounted for 0.09%. The 33 *env* loci ranged from 90.21%-99.70%. The remaining 31 *env* loci ranged from 13.57%-89.99%. Among all 76 proviral elements, 25 3’ LTR regions have been deleted. The longest 3’ LTR element accounted for 41.47%. The shortest 3’ LTR element accounted for 3.40%. The remaining 49 3’ LTR loci ranged from 6.32%-40.84%. In summary, 63 5’ LTRs, 43 *gag* regions, 34 *pro* regions, 19 *pol* regions, 11 *env* regions, and 25 3’ LTR regions have been completely deleted. The loss of the 5’ LTR was the most severe and was much greater than that of the 3’ LTR. The 5’ LTR plays a crucial role in the transcription and replication of viruses. Therefore, the consistent truncation of the HML-8 5’ LTRs likely significantly impedes their expression and retrotransposition activity in the chimpanzee genome. In contrast, the *env* region has the smallest degree of absence. Only 11 have been deleted. Forty-four out of the 76 *env* regions accounted for ≥70.75%. Interestingly, a similar situation was also observed in human genome, suggesting that HML-8 was integrated before the divergence of human and chimpanzee ancestors. In the human genome, among all 40 proviral elements, 28 5’ LTR regions have been deleted. The longest 5’ LTR accounted for 73.93% of the total length relative to the corresponding reference region. The shortest 5’ LTR accounted for 28.2%. The remaining 10 5’ LTRs ranged from 32.94%-73.14%. Among all 40 proviral elements, the *gag* regions of 17 have been deleted. The shortest *gag* accounts for 39.02%. The 12 *gag* regions ranged from 92.89%-99.95%. The remaining 10 gag regions ranged from 49.64%-81.41%. Among all 40 proviral elements, the *pro* region of 12 was deleted. There were 3 complete *pro* regions. The shortest *pro* accounted for 8.06%. The 12 *pro* regions ranged from 94.93%-99.5%. The remaining 12 ranged from 13.23%-88.46%. Among all 40 proviral elements, the *pol* region of 6 was deleted. The shortest *pol* accounted for 6.7%. The 10 *pol* regions ranged from 93.33%-99.89%. The remaining 23 *pol* regions ranged from 10.29%- 78.35%. Among all 40 proviral elements, the *env* region of 6 has been deleted. The shortest *env* gene accounted for 13.57%. The 15 *env* loci ranged from 90.16%-99.05%. The remaining 18 *env* loci ranged from 30.07%- 89.73%. Among all 40 proviral elements, the 3’ LTR region of 16 was missing. The longest 3’ LTR accounted for 75.36%. The shortest 3’ LTR accounted for 8.93%. The remaining 22 3’ LTRs ranged from 9.64%-44.71%. In summary, 28 5’ LTR regions, 17 *gag* regions, 12 *pro* regions, 6 *pol* regions, 6 *env* regions, and 16 3’ LTR regions were completely missing.

**Table 2:** The integrity of 6 separate regions relative to the corresponding sections of reference.

Number	Provirus Regions	5’LTR(%)	gag(%)	pro(%)	pol(%)	env(%)	3’LTR(%)
1	chr11 97063674 97072831	48.66%	99.67%	100.00%	99.71%	99.40%	40.36%
2	chr19 23582963 23597406	38.86%	99.43%	99.40%	97.66%	98.15%	37.12%
3	chr17 28556159 28565079	66.11%	99.29%	98.71%	97.25%	90.89%	32.86%
4	chr1 156345936 156354251	50.08%	72.83%	65.77%	95.20%	99.57%	36.41%
5	chr9 31695596 31703805	80.81%	99.72%	99.70%	99.67%	67.48%	0.00%
6	chr5 52655093 52662923	0.00%	92.79%	98.91%	99.45%	90.89%	30.65%
7	chr19 25615095 25622844	0.00%	93.22%	100.00%	97.07%	90.81%	28.99%
8	chr12 51714625 51722440	0.00%	93.12%	99.80%	99.74%	91.07%	28.99%
9	chr6 73941843 73949302	0.00%	85.44%	99.90%	99.60%	90.68%	15.01%
10	chr9 84591713 84599232	75.20%	99.67%	99.30%	30.92%	99.27%	36.97%
11	chr3 79615035 79622061	0.00%	93.65%	98.31%	65.97%	90.42%	41.47%
12	chrX 56602551 56609242	0.00%	91.80%	99.00%	99.16%	61.04%	0.00%
13	chr1 135696367 135712562	0.00%	36.65%	96.02%	99.23%	89.52%	35.23%
14	chr11 63656712 63663509	75.91%	70.22%	0.00%	57.33%	99.31%	9.64%
15	chr2A 64123614 64129736	0.00%	69.27%	77.51%	62.97%	90.51%	32.86%
16	chr3 128565266 128571536	0.00%	93.84%	51.14%	57.99%	91.19%	30.65%
17	chr12 81696811 81702819	0.00%	77.15%	56.02%	99.82%	54.90%	0.00%
18	chr11 49590235 49596119	0.00%	49.88%	99.90%	99.56%	59.66%	0.00%
19	chr10 98677603 98683109	50.00%	99.62%	25.97%	10.07%	98.71%	0.00%
20	chr1 108430301 108435591	76.54%	99.29%	98.81%	17.11%	39.65%	0.00%
21	chrY 23381750 23386956	0.00%	86.58%	78.61%	24.21%	95.83%	0.00%
22	chr11 50352478 50357771	0.00%	0.00%	14.93%	99.34%	90.64%	38.47%
23	chr11 49637737 49642530	0.00%	51.07%	99.80%	41.17%	65.42%	22.20%
24	chr4 137449286 137454131	33.49%	75.30%	0.00%	14.91%	88.53%	0.00%
25	chr12 102968149 102972730	0.00%	0.00%	0.00%	78.46%	91.15%	35.70%
26	chr3 109740319 109744759	0.00%	0.52%	82.19%	36.52%	99.70%	37.20%
27	chr11 14869711 14874159	0.00%	0.00%	33.33%	63.85%	91.07%	33.10%
28	chr4 64843173 64847502	0.00%	0.00%	0.00%	70.18%	99.61%	18.01%
29	chrX 34789238 34793597	0.00%	0.00%	16.02%	64.10%	90.85%	40.28%
30	chr8 44511870 44516437	14.14%	90.04%	99.60%	48.39%	0.00%	0.00%
31	chr6 155777868 155781847	0.00%	0.00%	0.00%	92.89%	67.65%	0.00%
32	chr19 24073629 24078094	0.00%	82.12%	99.80%	56.92%	0.00%	0.00%
33	chr4 77803811 77807756	0.00%	0.00%	55.72%	41.87%	90.46%	24.41%
34	chr8 12333790 12337817	0.00%	0.00%	0.00%	61.68%	90.59%	28.83%
35	chr7 6075204 6079128	0.00%	0.00%	0.00%	59.12%	90.94%	26.15%
36	chr8 43749874 43753733	0.00%	0.00%	34.03%	41.47%	90.34%	35.55%
37	chrY 6360429 6370254	0.00%	0.00%	34.03%	41.98%	89.99%	28.99%
38	chr8 86619640 86623356	0.00%	0.00%	3.28%	62.45%	70.75%	35.70%
39	chr20 29240803 29250628	0.00%	0.00%	34.03%	41.98%	89.99%	28.99%
40	chr4 77195305 77198763	0.00%	0.00%	0.00%	45.75%	99.01%	3.40%
41	chr2A 102337670 102341003	0.00%	0.00%	0.00%	37.29%	90.59%	27.33%
42	chr5 150548523 150551566	0.00%	0.00%	19.20%	63.77%	55.67%	0.00%
43	chr7 50863865 50867270	0.00%	0.00%	7.06%	64.03%	55.93%	0.00%
44	chr1 45743923 45746943	45.26%	99.43%	37.61%	0.00%	0.00%	0.00%
45	chrX 41627863 41630750	0.00%	0.00%	0.00%	17.62%	90.72%	33.81%
46	chrX 42154219 42156882	0.00%	0.00%	0.00%	6.96%	90.46%	38.63%
47	chr22 1446161 1448721	0.00%	86.39%	88.06%	0.00%	0.00%	0.00%
48	chr16 44723847 44726410	0.00%	0.00%	0.00%	5.13%	90.21%	35.86%
49	chrY 3095532 3098114	0.00%	0.00%	0.00%	6.59%	90.89%	33.18%
50	chrY 13438427 13440964	0.00%	0.00%	0.00%	6.92%	90.51%	29.62%
51	chr19 20847619 20850034	0.00%	37.55%	99.90%	31.21%	0.00%	0.00%
52	chr8 11324731 11327317	0.00%	0.00%	0.00%	8.86%	91.02%	28.75%
53	chr1 31825639 31827932	0.00%	0.00%	34.03%	42.01%	42.53%	0.00%
54	chr2B 5807936 5810201	0.00%	0.00%	0.00%	0.00%	77.15%	36.89%
55	chr4 49398166 49400489	0.00%	0.00%	0.00%	0.00%	80.37%	35.15%
56	chr15 19096168 19099630	0.00%	0.00%	0.00%	2.67%	82.04%	32.62%
57	chr4 162706670 162708848	0.00%	0.00%	0.00%	0.00%	77.32%	29.70%
58	chr2B 100074966 100077139	0.00%	0.00%	0.00%	0.00%	87.84%	9.95%
59	chr18 44459897 44461968	0.00%	0.00%	0.00%	0.00%	84.75%	6.32%
60	chr19 25500990 25503068	0.00%	84.87%	47.56%	0.00%	0.00%	0.00%
61	chr14 71784651 71786596	0.00%	0.00%	0.00%	0.00%	61.25%	40.84%
62	chr4 55246241 55248103	0.00%	0.00%	33.93%	57.33%	0.00%	0.00%
63	chr6 58293089 58294835	0.00%	5.22%	91.84%	31.61%	0.00%	0.00%
64	chr13 59301174 59302916	0.00%	0.00%	0.00%	0.00%	53.91%	37.28%
65	chr11 49783110 49784802	0.00%	0.00%	0.00%	61.94%	0.09%	0.00%
66	chr2A 77283713 77285331	0.00%	76.72%	0.00%	0.00%	0.00%	0.00%
67	chr2B 97034956 97036587	0.00%	0.00%	0.00%	0.00%	54.17%	28.83%
68	chr5_NW_019932883v1_random 1485086 1486708	48.97%	39.26%	0.00%	0.00%	0.00%	0.00%
69	chr4 50892584 50893971	0.00%	0.00%	0.00%	0.00%	54.25%	9.56%
70	chrY 14091617 14092838	0.00%	0.00%	0.00%	0.00%	41.92%	19.04%
71	chrY 5451943 5453164	0.00%	0.00%	0.00%	0.00%	41.92%	19.04%
72	chrX 46463406 46464551	0.00%	0.00%	34.03%	30.99%	0.00%	0.00%
73	chrY 1087726 1088806	0.00%	0.00%	0.00%	0.00%	41.92%	8.06%
74	chr5 32949251 32950412	0.00%	0.00%	0.00%	0.00%	30.03%	35.70%
75	chr10 77164804 77165954	0.00%	0.00%	0.00%	0.00%	26.63%	39.89%
76	chr1 104828192 104829066	0.00%	0.00%	0.00%	25.68%	13.57%	0.00%

### 3.3 Phylogenetic analyses

**Figure 4:**
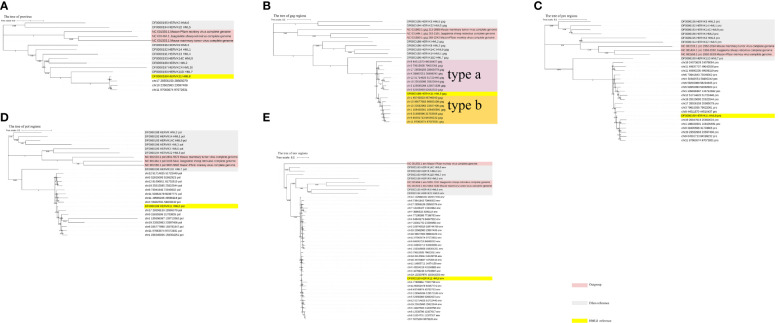
Phylogenetic analysis of the HML-8 near-full-length proviruses and 4 subregions by the maximum likelihood method. Phylogenetic analyses of 3 HML-8 proviral elements **(A)**, 15 *gag* elements **(B)**, 19 *pro* elements **(C)**, 15 *pol* elements **(D)**, and 25 *env* elements **(E)**, along with reference sequences. The generated phylogenetic trees were all tested by the bootstrap method with 500 replicates. The branch length indicates the number of substitutions per site. The two intragroup clusters of the *gag* genes (types a and b) were annotated and depicted with different colors, respectively.

To further confirm the assignment of identified HML-8 elements in the chimpanzee genome and characterize their phylogenetic relationships, an ML phylogenetic tree for near-full-length proviruses was first constructed. Three proviral sequences (longer than 80% of the HML-8 reference length) were screened to generate their phylogenetic relationships (**Figure 4A**). Next, 4 ML trees were constructed for subregions whose lengths were longer than 90% of the corresponding section of the reference sequence; these included 15 *gag* elements, 19 *pro* elements, 15 *pol* elements, and 33 *env* elements (**Figures 4B–E**). For comparison, the Dfam HERV-K group (HML-1–10) and 3 exogenous betaretroviruses were used as representatives and outgroups, respectively. These phylogenetic groups of different regions of HML-8 were all distinctly separated from the other HERV-K groups (HML1-7, 9-10) (**Figures 4A-E**). The 3 screened proviruses all clustered with the Dfam HML-8 reference supported by bootstrap support of 100%, indicating that they significantly more likely to be HML-8 than any other HML subtypes (**Figure 4A**). The phylogenetic groups for different regions of HML-8 all clustered together with their corresponding sections of the HML-8 reference, respectively (bootstrap support of 100% for *gag*, *pol*, and *pro*, 92.2% for *env*). Interestingly, two distinct clusters in the *gag* group were identified. The strains were statistically supported by ≥95% of bootstrap values and were named HML-8 type a and type b. The results showed that chr8 44511870 44516437, chr3 79615035 79622061, chr17 28556159 28565079, chrX 56602551 56609242, chr12 51714625 51722440, chr19 25615095 25622844, chr3 128565266 128571536, and chr5 52655093 52662923 were included in type a, whereas chr1 45743923 45746943, chr10 98677603 98683109, chr19 23582963 23597406, chr1 108430301 108435591, chr9 31695596 31703805, chr9 84591713 84599232, and chr11 97063674 97072831 were included in type b. HML-8 type b sequences included the Dfam HML-8 reference, whereas HML-8 type a elements showed more divergence relative to the HML-8 reference. There are no solo LTRs in the chimpanzee genome. Thus, no phylogenetic trees for solo LTRs have been constructed.

### 3.4 Estimated time of integration

Like the distribution dynamics and other characteristics of these remnants, the integration time of each chimpanzee HML-8 member is also a key clue to understanding the evolution of the group across primates. Given the serious lack of intact LTRs of the proviruses, i.e., no provirus has a 5’ LTR or 3’ LTR greater than 70%, the proviral LTRs were not used for the integration time calculation as previously described (Jia et al., 2022; Liu et al., 2023). Here, we estimated the age of the 46 HML-8 proviral elements in the chimpanzee genome based on the available *gag*, *pro*, *pol*, and *env* regions, respectively (**Table 3**). Each region whose length exceeds 90% of the corresponding reference sequence was used to calculate the integration time. Through the formula, an estimate of the integration time (T) can be obtained, namely, T = D/0.2, where D is the percentage of divergent nucleotides and 0.2 represents the host genome neutral mutation rate expressed in substitutions/nucleotide/million years. For each proviral region mentioned above, the ancestral sequences of each region were generated via MEGA7 based on multiple alignments of all the elements and the ML method. The details of the proviral formation periods are shown in **Table 3**. Overall, the HML-8 elements (*gag*, *pro*, *pol*, and *env*) found in the chimpanzee genome were integrated between 15 and 52.33 million years ago (mya). The average integration time was 35.86 mya, and the median was 37.25 mya. In our previous study, we performed a comprehensive identification and characterization of the HML-8 group in the human genome (Liu et al., 2023). Through comparison, it was found that the integration of human HML-8 elements mainly occurred between 23.5 and 52 mya. The average integration time was 37.11 mya, and the median was 37.42 mya. The divergence between human and chimpanzee ancestors is known to trace back to approximately 6.5–7.5 mya or earlier. The results indicated that the chimpanzee-specific insertion periods were indeed similar to the human-specific insertion periods and further confirmed that HML-8 was integrated into common ancestors before humans and chimpanzees diverged.

**Table 3:** Estimated time of HML-8 elements integration.

Number	Provirus regions	Divergence from Consensus sequence				Mean Divergences	T = D/0.2	Age/ million years (gene vs consensus)
		gag	pro	pol	env			
1	chr11 97063674 97072831	0.113	0.087	0.086	0.076	0.091	0.4525	45.25
2	chr19 23582963 23597406	0.109	0.075	0.096	0.087	0.092	0.45875	45.875
3	chr17 28556159 28565079	0.064	0.062	0.077	0.054	0.064	0.32125	32.125
4	chr1 156345936 156354251	NA	NA	0.092	0.070	0.081	0.405	40.5
5	chr9 31695596 31703805	0.124	0.089	0.101	NA	0.105	0.523333333	52.33333333
6	chr5 52655093 52662923	0.100	0.050	0.029	0.054	0.058	0.29125	29.125
7	chr19 25615095 25622844	0.099	0.071	0.039	0.068	0.069	0.34625	34.625
8	chr12 51714625 51722440	0.129	0.080	0.052	0.063	0.081	0.405	40.5
9	chr6 73941843 73949302	NA	0.050	0.075	0.054	0.060	0.298333333	29.83333333
10	chr9 84591713 84599232	0.109	0.091	NA	0.074	0.091	0.456666667	45.66666667
11	chr3 79615035 79622061	0.048	0.054	NA	0.047	0.050	0.248333333	24.83333333
12	chrX 56602551 56609242	0.068	0.059	0.065	NA	0.064	0.32	32
13	chr1 135696367 135712562	NA	0.071	0.044	NA	0.058	0.2875	28.75
14	chr11 63656712 63663509	NA	NA	NA	0.075	0.075	0.375	37.5
15	chr2A 64123614 64129736	NA	NA	NA	0.045	0.045	0.225	22.5
16	chr3 128565266 128571536	0.088	NA	NA	0.043	0.066	0.3275	32.75
17	chr12 81696811 81702819	NA	NA	0.041	NA	0.041	0.205	20.5
18	chr11 49590235 49596119	NA	0.082	0.085	NA	0.084	0.4175	41.75
19	chr10 98677603 98683109	0.099	NA	NA	0.069	0.084	0.42	42
20	chr1 108430301 108435591	0.107	0.078	NA	NA	0.093	0.4625	46.25
21	chrY 23381750 23386956	NA	NA	NA	0.098	0.098	0.49	49
22	chr11 50352478 50357771	NA	NA	0.078	0.065	0.072	0.3575	35.75
23	chr11 49637737 49642530	NA	0.085	NA	NA	0.085	0.425	42.5
25	chr12 102968149 102972730	NA	NA	NA	0.031	0.031	0.155	15.5
26	chr3 109740319 109744759	NA	NA	NA	0.078	0.078	0.39	39
27	chr11 14869711 14874159	NA	NA	NA	0.049	0.049	0.245	24.5
28	chr4 64843173 64847502	NA	NA	NA	0.062	0.062	0.31	31
29	chrX 34789238 34793597	NA	NA	NA	0.040	0.040	0.2	20
30	chr8 44511870 44516437	0.105	0.075	NA	NA	0.090	0.45	45
31	chr6 155777868 155781847	NA	NA	0.093	NA	0.093	0.465	46.5
32	chr19 24073629 24078094	NA	0.076	NA	NA	0.076	0.38	38
33	chr4 77803811 77807756	NA	NA	NA	0.075	0.075	0.375	37.5
34	chr8 12333790 12337817	NA	NA	NA	0.065	0.065	0.325	32.5
35	chr7 6075204 6079128	NA	NA	NA	0.066	0.066	0.33	33
36	chr8 43749874 43753733	NA	NA	NA	0.087	0.087	0.435	43.5
40	chr4 77195305 77198763	NA	NA	NA	0.084	0.084	0.42	42
41	chr2A 102337670 102341003	NA	NA	NA	0.030	0.030	0.15	15
44	chr1 45743923 45746943	0.083	NA	NA	NA	0.083	0.415	41.5
45	chrX 41627863 41630750	NA	NA	NA	0.074	0.074	0.37	37
46	chrX 42154219 42156882	NA	NA	NA	0.046	0.046	0.23	23
48	chr16 44723847 44726410	NA	NA	NA	0.054	0.054	0.27	27
49	chrY 3095532 3098114	NA	NA	NA	0.090	0.090	0.45	45
50	chrY 13438427 13440964	NA	NA	NA	0.095	0.095	0.475	47.5
51	chr19 20847619 20850034	NA	0.091	NA	NA	0.091	0.455	45.5
52	chr8 11324731 11327317	NA	NA	NA	0.068	0.068	0.34	34
63	chr6 58293089 58294835	NA	0.068	NA	NA	0.068	0.34	34

“NA” stands for Not Applicable.

Despite all this, there are significant differences in distribution quantity and structural form. The chimpanzees included 76 HML-8 proviral elements and 0 solo LTRs. By comparison, there are only 40 proviruses in the human genome, almost half as many as in the chimpanzee genome. In addition, the human genome also contains 5 solo LTRs. Solo LTRs arise from recombination between LTRs and the removal of intermediate regions of a provirus, and these recombination events mainly occur during meiotic recombination (Jia and Li, 2018). This significant difference precisely indicated that even after integration, the interaction between the pathogen and its host did not stop. The host genome can retain helpful or select against harmful proviral integrations. The chimpanzee genome contains more HML-8 proviral elements (76 vs. 40) and fewer solo LTRs (0 vs. 5) than humans. Since HML-8 integration occurred more than 30 million years prior to the divergence of chimpanzees and humans, the different distribution and number of these elements is likely due to differences in selection on these proviruses in the different species. This suggests that HML-8 integrations were retained at a greater rate in the chimpanzee genome than in the human genome, perhaps due to selection pressure differences or different rates of recombination during meiosis. Our results may suggest the difference in genome response to proviral integration contributed to the speciation event, which created humans and chimpanzees as distinct species.

### 3.5 Functional prediction of cis-regulatory regions and enrichment analysis

**Figure 5:**
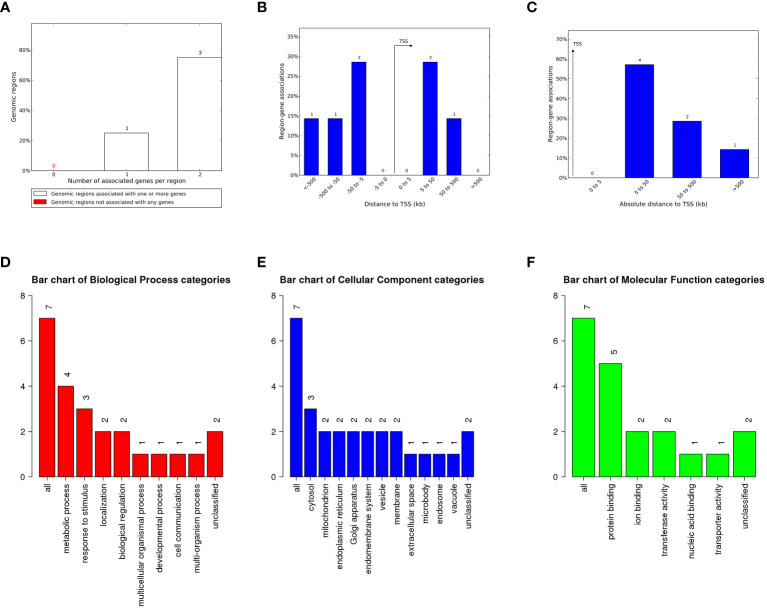
The genes associated with proviral LTRs and GO Slim summaries. **(A)** The number of associated genes per proviral LTR. **(B)** Binned by orientation and distance to the TSS. **(C)** Binned by the absolute distance to the TSS. The biological process **(D)**, cellular component **(E)**, and molecular function **(F)** categories are represented by red, blue, and green bars, respectively. The height of the bar represents the number of IDs in the gene list and in the category.

The LTR plays a crucial role in virus transcription and replication. Although most HML-8 LTRs are severely truncated, any regulatory sites present in the remaining sequence can play a role in the host genome’s functional process as cis-regulatory regions. The tool of Genomic Regions Enrichment of Annotations Tool (GREAT) can predict the biological significance of these noncoding regions by analyzing annotations of nearby genes, i.e., based on spatial proximity. For the chimpanzee-specific HML-8 proviral LTRs, we selected LTR sequences larger than 70% of the reference sequence for further prediction. The results describing the associations between each proviral LTR and its putative-regulated gene(s) are shown in **Supplementary Table S1**. Seven genes were predicted in total. Among them, 1 LTR was associated with 1 gene, and 3 LTRs were associated with 2 genes (**Figure 5A**; **Supplementary Table S1**). No gene had an absolute distance from the transcription start site (TSS) of less than 5 kb. The absolute distance basically measures how far the gene is from the TSS. The absolute distances between the 4 genes and the TSS were 5 to 50 kb. The absolute distance between the 2 genes and the TSS was between 50 and 500 kb. The absolute distance between 1 gene and its TSS was greater than 500 kb (**Figures 5B, C**). To analyze the biological taxonomy of genes associated with LTRs, we produced GO Slim summaries to annotate these genes to functional categories. GO biological process (BP) analysis revealed that these genes were involved mainly in metabolic processes, responses to stimulus, localization, and biological regulation (**Figure 5D**). The GO Slim cellular component (CC) summary showed that these genes were significantly involved in the cytosol, mitochondrion, and endoplasmic reticulum, and the GO Slim molecular function (MF) summary revealed that these genes were significantly involved in protein binding, ion binding, and transferase activity (**Figures 5E, F**).

**Figure 6:**
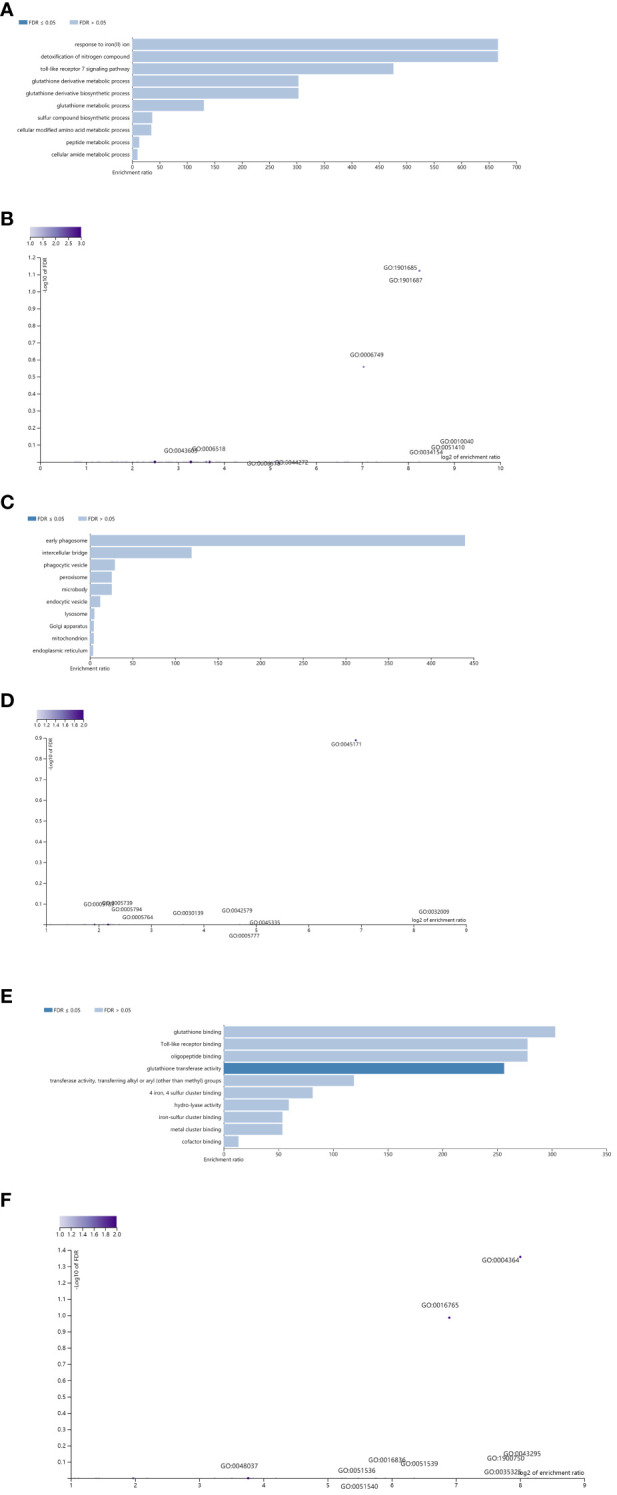
Enrichment result categories binned by biological process, cellular component, and molecular function. **(A, B)** Bar chart and customizable volcano plot of the biological process enrichment results. A bar graph showing the enrichment ratio of the results was constructed. Bars representing categories with an FDR ≤ 0.05 are shown in a darker shade **(A)**. The volcano plot in **(B)** shows the log2 of the FDR versus the enrichment ratio for all the functional categories in the database, highlighting the degree to which the significant categories are separated from the background. The size and color of a dot are proportional to the number of overlaps (for ORA). The significantly enriched categories are labeled, and the labels are positioned automatically by a force field-based algorithm at startup. **(C, D)** Bar chart and customizable volcano plot of the cellular component enrichment results. **(E, F)** Bar chart and customizable volcano plot of the molecular function enrichment results.

Moreover, these potential regulatory genes were subjected to enrichment analysis using WebGestalt. The top 10 most significant GO terms according to the FDR value for BPs included “response to iron(II)ion”, “detoxification of nitrogen compound”, “toll-like receptor 7 signaling pathway”, “glutathione derivative metabolic process”, “glutathione metabolic process”, “sulfur compound biosynthetic process”, “cellular modified amino acid metabolic process”, “peptide metabolic process”, and “cellular amide metabolic process” (**Figure 6A**).

The enrichment results for the CC and MF categories are shown in **Figures 6C–F**. As repeatedly emphasized in our previous papers, all these results are entirely prediction-based, and future biological research is needed to confirm any of the implied associations between proviral LTRs and nearby genes.

## 4 Discussion

ERV is an indispensable partner in the evolutionary process of primates. The integration and coevolution of ERVs can shape the host genome and participate in physiological and pathological processes (Johnson, 2019; Jansz and Faulkner, 2021; Chen et al., 2022). Therefore, it is critical to study the distribution of HML-8 loci in the chimpanzee genome to understand their evolutionary history and to inform future functional research. Previously, we conducted a comprehensive identification and characterization of the HML-8 group in the human genome (Liu et al., 2023). However, there is still a lack of comprehensive understanding of the evolutionary history of ERVs in other primates; for example, chimpanzees, which are the closest living genetic relatives to humans and share much of our genetic information, including ERV integrated in the genome. The distribution and function prediction of HML-8 in chimpanzees remain unclear and thus the comparisons of these elements between the two hosts cannot be carried out. We further characterized these remnants in chimpanzees and provided a detailed description of the HML-8 proviruses in the chimpanzee genome, including the HML-8 genome distribution, structural characteristics, phylogeny, integration time analysis, and regulatory function prediction.

We identified a total of 76 HML-8 proviral elements, and the results showed that the distribution of these proviral elements in the chimpanzee genome was nonrandom. Our previous studies have shown that the distribution of HML-8 loci in humans is not random (p<0.005). Our comparison between HML-8 elements in the human and chimpanzee genomes showed that there is great similarity in the distribution of proviral chromosomal positions between chimpanzees and humans. Both genomes showed significant enrichment of proviral integration in the 11, 19, and Y chromosomes of chimpanzees compared to the predicted number.

Like in humans, the number of proviral elements integrated into the Y chromosome of chimpanzees was significantly greater than that predicted (p<0.05). The Y chromosome is one of the two sex chromosomes that determines male sex. It not only is structurally complex but also the fastest-changing chromosome among human chromosomes. In addition to features related to sex determination, genes on the Y chromosome also have an impact on other traits and diseases in humans, such as the risk and severity of cancer (Rhie et al., 2023). There are several possible reasons for the insertions into the Y chromosome. The first possibility for additional provirus insertions may be due to the gene density on the Y chromosome, which became fixed in the population due to a decreased chance of gene disruption. An insertion on the Y chromosome may have a lower chance of being deleterious and, therefore, would be more likely to be retained and passed on to the next generation. In addition, the physical placement of the chromosome within the nucleus and the chromatin status also strongly influence whether a provirus can be inserted into that portion of the genome (Rhie et al., 2023). Anyhow, ERV enrichment on the Y chromosome could suggest that these elements may be deeply involved in reproduction, disease, and other unresolved processes.

Structural characterization revealed that no HML-8 members retained near full-length proviral structures. All the HML-8 elements have become fragmented due to insertion, deletion, or other mutations during the long history of evolution, including a total of 63 complete deletions of the 5’ LTR sequence and 25 complete deletions of the 3’ LTR of the proviruses. The middle four open reading frames (*gag*, *pro*, *pol*, and *env*) had 43, 34, 19, and 11 complete deletions, respectively. Such a large-scale deficiency reflects the host’s ability to reshape foreign elements, screening out harmful elements and leaving behind useful elements. Subregion phylogenetic analysis of 4 internal regions revealed that 15 *gag* elements, 19 *pro* elements, 15 *pol* elements, and 33 *env* sequences formed a unique cluster, each of which was supported by strong bootstrap values, confirming their assignment with great certainty.

The integration time of most HML-8 elements (*gag*, *pro*, *pol*, and *env*) found in the chimpanzee genome is mainly between 15 and 52.33 mya, with an average integration time of 35.86 mya and a median of 37.25 mya, which are very similar to those of humans. These results further confirmed that HML-8 was integrated before the divergence between human and chimpanzee ancestors, which occurred approximately 6.5–7.5 mya ago. The integration and coevolution of ERVs can reshape the host genome and participate in physiological and pathological processes (Johnson, 2019; Jansz and Faulkner, 2021; Chen et al., 2022). The significant differences in quantity and structure of HML-8 between humans and chimpanzees obtained from the present study indicated that, in turn, the host will also screen and reshape the external elements integrated from the outside. Even after proviral integration has completed, interactions between the host genome and the inserted provirus continue. Integrated exogenous retroviruses will undergo genetic recombination according to the evolutionary mechanisms of the host genome following meiotic recombination, site-specific recombination, and transpositional recombination (Jia et al., 2016; Jia and Li, 2018). A typical remnant of the original and complete provirus is solo LTR which arise from host homologous recombination between ancestral 5’ and 3’ proviral LTRs, where the intervening protein-coding sequence is deleted (Mager and Goodchild, 1989; Hughes and Coffin, 2004; Jia and Li, 2018; Thomas et al., 2018). It was reported that at least 85% of reference genome ERV instances are solo LTRs (Lander et al., 2001; Mager and Stoye, 2015; Thomas et al., 2018). Compared to humans, chimpanzees maintain many more proviral elements and fewer solo LTRs, indicating that the active interaction between the chimpanzee genome and the integrated proviruses is lower than that of the human genome which has a greater ability to shape integrated proviral elements.

In summary, we have described in detail the existence and distribution of HML-8 elements in the chimpanzee genome, as well as the structural characterization and phylogenetic analysis of these remnants. In addition, we further predicted the potential biological function of the genes related to proviral LTRs via bioinformatics methods. Our work revealed that the chimpanzee genome contains fewer chimpanzee-specific HML-8 solo LTR integration but more chimpanzee-specific HML-8 provirus integration, suggesting that HML-8 elements evolved in different ways after the divergence of human and chimpanzee ancestors. The results of the present study could provide a comprehensive research background for the differences between human and chimpanzee genomes and the potential implications in the future.

## Data availability statement

The datasets presented in this study can be found in online repositories. The names of the repository/repositories and accession number(s) can be found in the article/**Supplementary Material**.

## Author contributions

CW: Writing – original draft, Data curation, Formal analysis. XZ: Writing – original draft, Formal analysis, Methodology. SW: Writing – original draft, Data curation, Methodology. BZ: Software, Writing – review & editing. CY: Software, Writing – review & editing. YS: Validation, Writing – review & editing. HL: Writing – review & editing, Validation. YL: Writing – review & editing, Validation. JH: Writing – review & editing, Visualization. XW: Writing – review & editing, Visualization. JL: Writing – review & editing, Visualization. MC: Writing – review & editing, Data curation, Methodology. LJ: Writing – review & editing, Conceptualization, Data curation, Formal analysis, Methodology, Writing – original draft. LL: Conceptualization, Writing – review & editing, Data curation, Formal analysis, Writing – original draft.
